# Butyrate production in the acetogen *Eubacterium limosum* is dependent on the carbon and energy source

**DOI:** 10.1111/1751-7915.13779

**Published:** 2021-02-25

**Authors:** Dennis Litty, Volker Müller

**Affiliations:** ^1^ Department of Molecular Microbiology & Bioenergetics Institute of Molecular Biosciences Goethe‐University Frankfurt am Main Hessen Germany

## Abstract

*Eubacterium limosum* KIST612 is one of the few acetogenic bacteria that has the genes encoding for butyrate synthesis from acetyl‐CoA, and indeed, *E. limosum* KIST612 is known to produce butyrate from CO but not from H_2_ + CO_2_. Butyrate production from CO was only seen in bioreactors with cell recycling or in batch cultures with addition of acetate. Here, we present detailed study on growth of *E. limosum* KIST612 on different carbon and energy sources with the goal, to find other substrates that lead to butyrate formation. Batch fermentations in serum bottles revealed that acetate was the major product under all conditions investigated. Butyrate formation from the C1 compounds carbon dioxide and hydrogen, carbon monoxide or formate was not observed. However, growth on glucose led to butyrate formation, but only in the stationary growth phase. A maximum of 4.3 mM butyrate was observed, corresponding to a butyrate:glucose ratio of 0.21:1 and a butyrate:acetate ratio of 0.14:1. Interestingly, growth on the C1 substrate methanol also led to butyrate formation in the stationary growth phase with a butyrate:methanol ratio of 0.17:1 and a butyrate:acetate ratio of 0.33:1. Since methanol can be produced chemically from carbon dioxide, this offers the possibility for a combined chemical‐biochemical production of butyrate from H_2_ + CO_2_ using this acetogenic biocatalyst. With the advent of genetic methods in acetogens, butanol production from methanol maybe possible as well.

## Graphical abstract

In this study, we have investigated the growth of and product formation by the acetogen *Eubacterium limosum* KIST612 on different carbon and energy sources with the goal to find substrates that lead to formation of butyrate. Butyrate is an important product that can be further reduced to the highly valuable biofuel butanol in a two‐step enzymatic process.

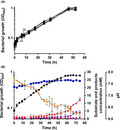

## Introduction

Acetogens are a physiological group of strictly anaerobic bacteria that are characterized by a special pathway for CO_2_ fixation, the Wood–Ljungdahl pathway (WLP) (Müller, 2003; Drake *et al*., 2008; Ragsdale, 2008). The WLP is a branched linear pathway in which two mol of CO_2_ are reduced to one mol of acetyl‐CoA which is further converted to acetate in all species under most conditions (Müller and Frerichs, 2013). Moreover, some species can convert acetyl‐CoA (or acetate) to ethanol or even to C4 compounds such as butyrate (Daniell *et al*., 2012; Jeong *et al*., 2015; Bengelsdorf *et al*., 2016). Therefore, acetogens have come into focus as biocatalysts for a CO_2_‐based bioeconomy and ethanol is already produced on an industrial scale using *Clostridium autoethanogenum* (Bengelsdorf *et al*., 2018; Heffernan *et al*., 2020). The addition of one carbon to the chain length of the product increases the value of the product by a factor of 1.5–3, depending on the product (Kim *et al*., 2019). Butyrate is not a prime product to be produced but it can be reduced to butanol in a two‐step enzymatic process and butanol is a highly desired biofuel (Dürre, 2016). Butyrate is produced naturally by only a few acetogens such as *Clostridium carboxidivorans* (Liou *et al*., 2005), *Clostridium drakei* (Küsel *et al*., 2000; Liou *et al*., 2005), *Oxobacter pfennigii* (Krumholz and Bryant, 1985) and *E. limosum* strains such as KIST612 (Pacaud *et al*., 1985; Loubiere and Lindley, 1991; Chang *et al*., 1997). The latter has gained much interest for it produces butyrate form synthesis gas (syngas), a mixture of H_2_, CO_2_ and CO in different concentrations, depending on the source (Chang *et al*., 2001; Park *et al*., 2017). Syngas is an industrial waste stream that is already been used as feedstock for acetogenic conversion to ethanol (Dürre and Eikmanns, 2015; Humphreys and Minton, 2018). However, butyrate formation in *E. limosum* KIST612 was only observed in bioreactors with cell recycling (Chang *et al*., 2001), but not in batch cultures or only to minor amounts when acetate was added to the culture (Park *et al*., 2017). Methanol and formate are two promising, alternative feedstocks for the industrial production of biofuels using acetogens as they can be produced from many sustainable feedstocks including biomass, municipial solid waste, biogas as well as CO_2_. One major advantage using these two feedstocks is that they are fully soluble and therefore can overcome the challenges gaseous C1 feedstocks are facing due to their low mass transfer. In addition, formate and methanol can also be easily transported and stored.

**Fig. 1:**
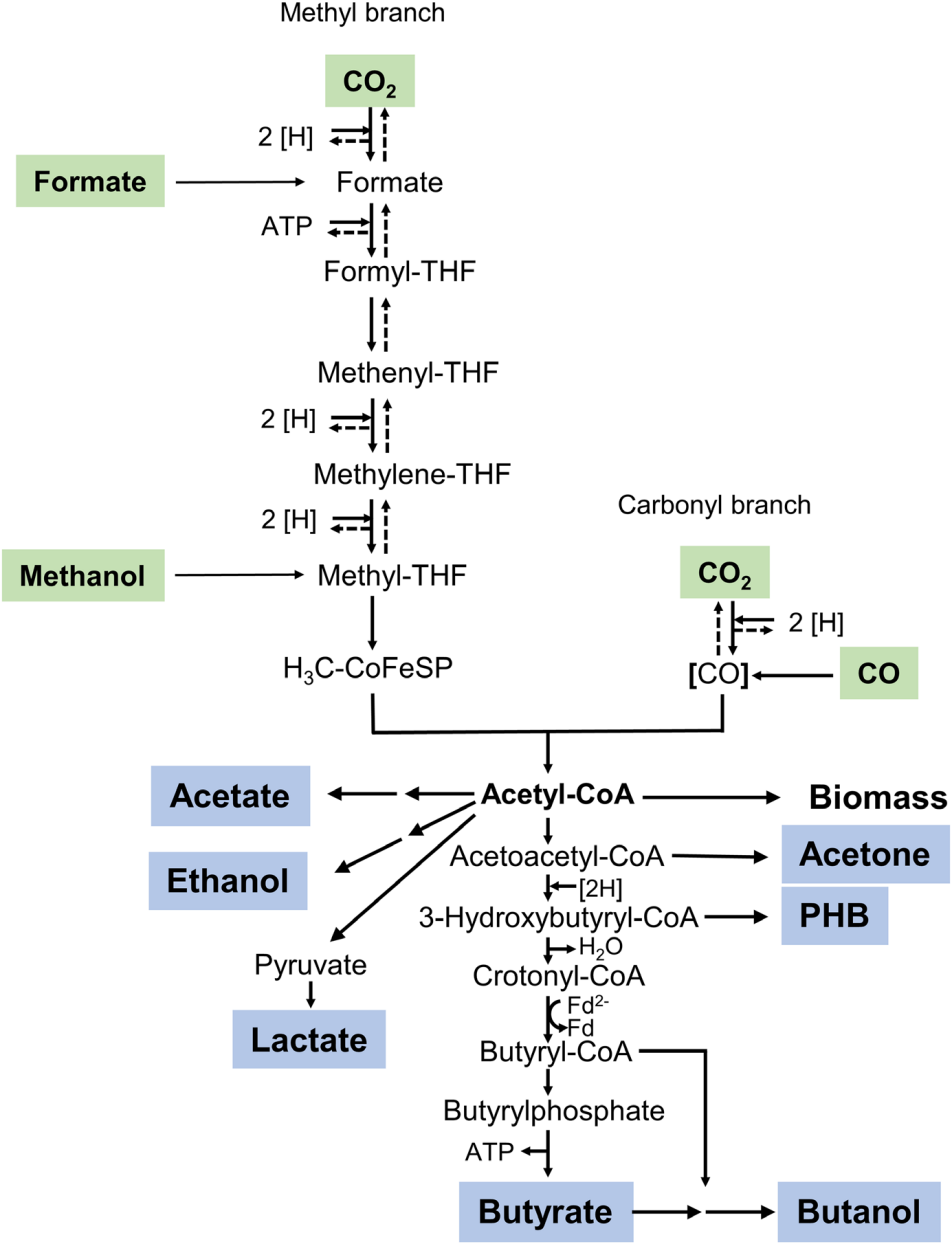
The Wood–Ljungdahl pathway of CO_2_ reduction. In the WLP, two molecules of CO_2_ are reduced to the central intermediate acetyl‐CoA. Entry points for other C1 substrates are indicated. Acetyl‐CoA is the precursor of biomass and a wide range of natural products (blue). The pathways leading from acetyl‐CoA to products are not complete and miss intermediates, reducing equivalents and ATP input/output. Only the pathway leading to butyrate is complete. CoFeSP, corrinoid/iron sulfur protein; THF, tetrahydrofolate; CoA, coenzyme A; [H], reducing equivalent.

The methyl group of methanol is channelled into the WLP by a methyltransferase system (Kremp *et al*., 2018; Kremp and Müller, 2020) whereas formate is an intermediate of the pathway (Fig. 1). Unfortunately, it is not known whether these C1 substrates maybe converted to butyrate as well. Here, we have investigated the physiology of growth of *E. limosum* KIST612 on different substrates with a focus on the production of butyrate.

## Results and discussion

### Growth with and product formation from glucose

**Fig. 2:**
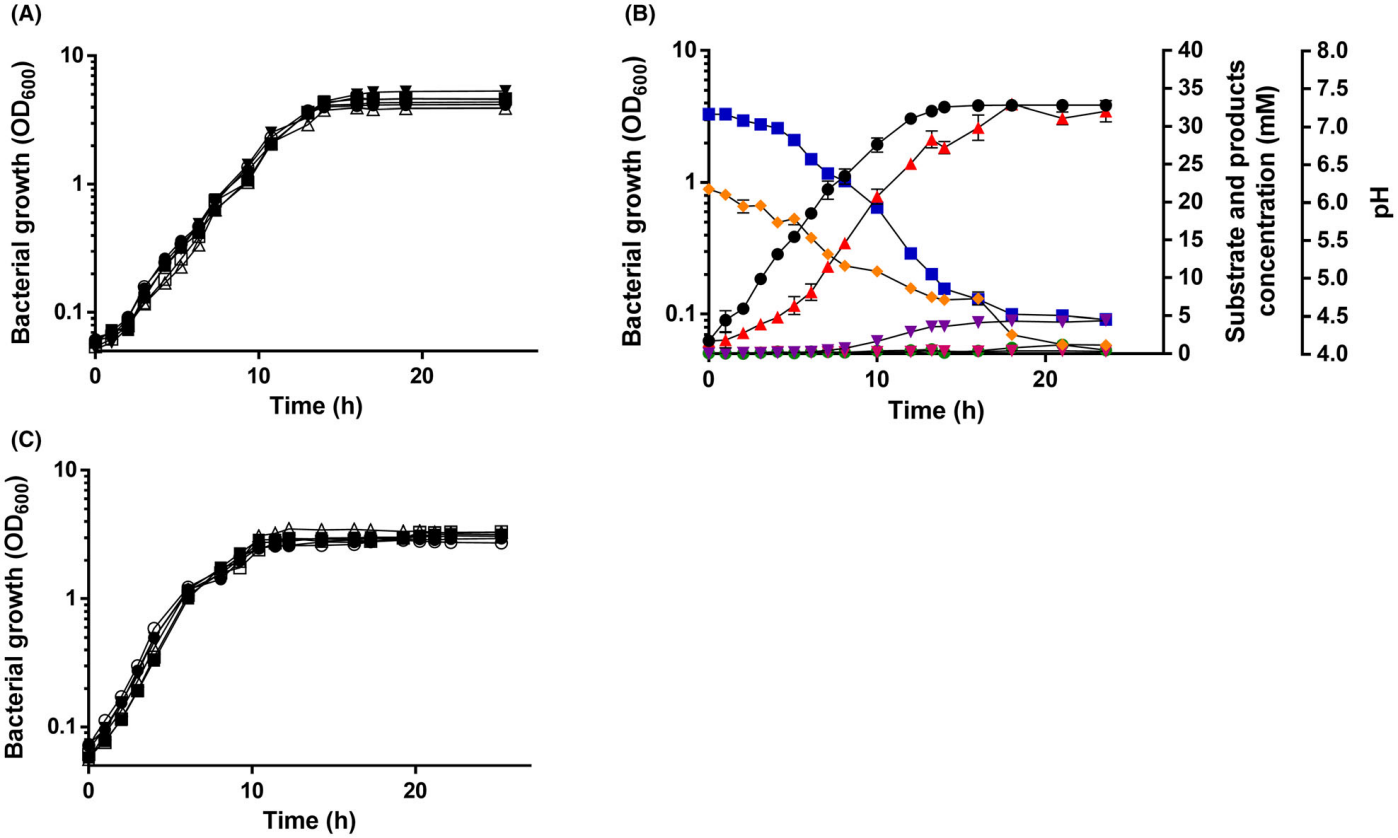
Growth of *E*. *limosum* on glucose and fructose. *E. limosum* KIST612 was grown at 37°C in 500 ml of carbonate‐buffered basal medium (CBBM) (Chang *et al*., 1999) with glucose under a N_2_/CO_2_ (80/20% [v/v]) atmosphere. Growth was followed by measuring the optical density (OD) at 600 nm. A. Growth of *E*. *limosum* on 20 (●), 40 (○), 80 (▼), 120 (▵), 160 (■) or 200 (□) mM glucose. B. Growth and product portfolio of *E*. *limosum* on 20 mM glucose. OD_600_ (●) was determined photometrically, pH (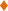) was determined with an Orion Basic electrode (Thermo Electron Corp. Witchford, UK). The concentration of glucose (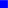) was determined by a d‐glucose/d‐fructose assay kit (R‐Biopharm, Pfungstadt, Germany). Acetate (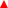), butyrate (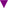) and ethanol (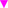) were measured by gas chromatography as described previously (Jeong *et al*., 2015). C. Growth of *E*. *limosum* on 20 (●), 40 (○), 80 (▼), 120 (▵), 160 (■) or 200 (□) mM fructose. All data points are mean ± SEM; *N* = 3 independent experiments.

When *E. limosum* KIST612 was transferred to carbonate buffered basal medium (CBBM) (Chang *et al*., 1999) containing different amounts of glucose ranging from 20 to 200 mM, growth rates were identical (*µ* = 0.34 h^−1^) as were the final yields (OD_600_ = 4.5). To describe the growth physiology in more detail, cells were cultured on 20 mM glucose. On transfer of a glucose‐adapted preculture to fresh medium, growth started immediately with a rate of 0.34 h^−1^ and proceeded for about 13 h, before the stationary phase started (Fig. 2). Parallel to growth, the glucose concentration dropped continuously with a rate of 1.4 mmol l^−1^∙h^−1^ to a residual concentration of 1.2 mM. In parallel, the pH dropped from 7.2 to 4.5. Glucose consumption was paralleled by a production of acetate that reached a final concentration of 32.1 mM, corresponding to an acetate:glucose ratio of 1.7:1. Interestingly, at the end of the exponential growth phase butyrate was produced and butyrate production reached a steady state at around 17 h. The final butyrate concentration was 4.3 mM, corresponding to a butyrate:glucose ratio of 0.23:1. Even later, at around 13 h, ethanol formation started, but ethanol formation was very small with values in the 0.4–0.7 mM range. In total, the recovery of glucose in all end products was 75 ± 3.9 %, not accounting for CO_2_. Growth on fructose led to similar growth characteristics (Fig. 2).

### Growth with and product formation from H_2_ + CO_2_, CO or formate

**Fig. 3:**
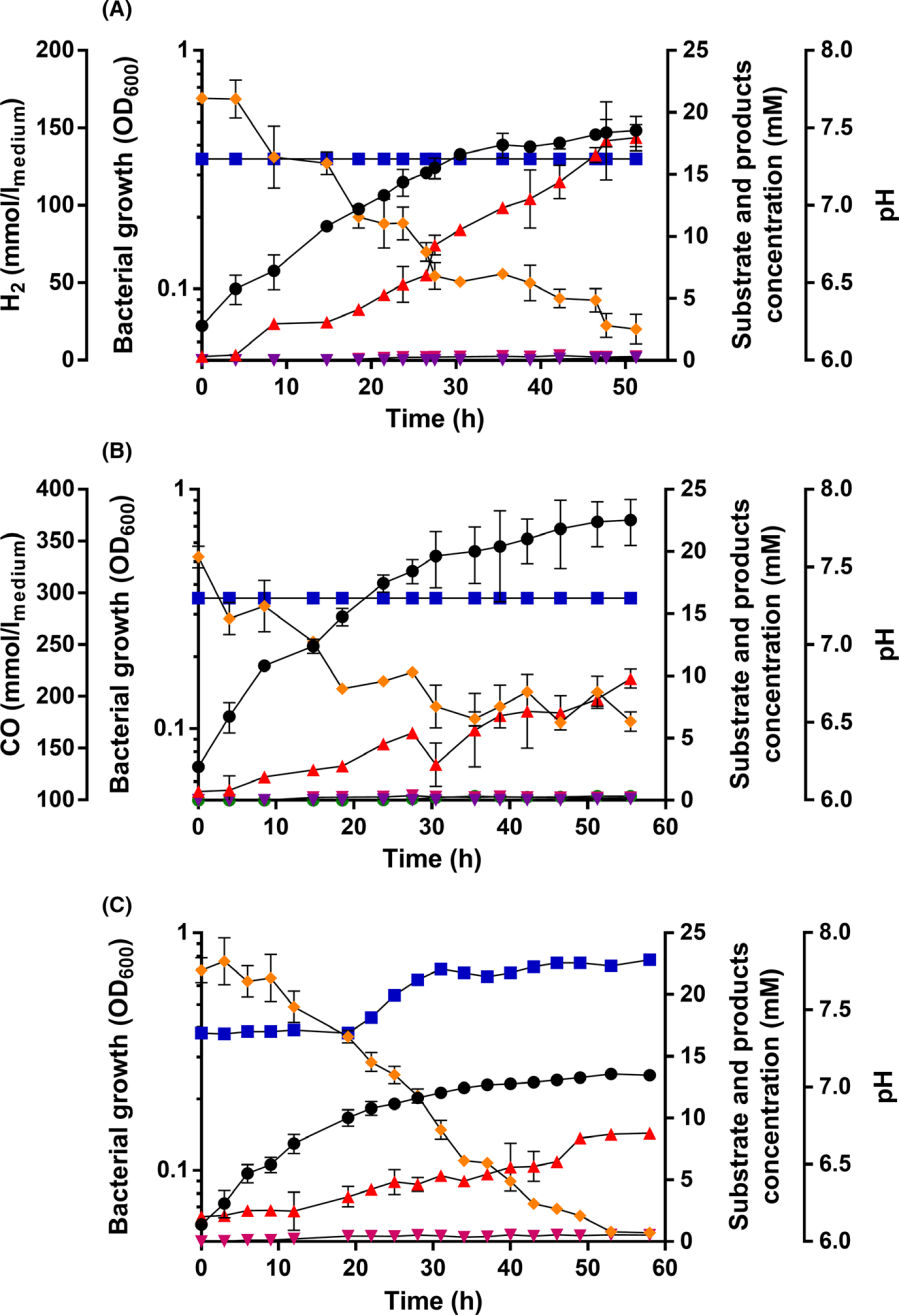
Growth of *E*. *limosum* on H_2_ + CO_2_, CO or formate. A. *E. limosum* was grown at 37°C in 500 ml of CBBM with overpressure of 1 bar H_2_ + CO_2_ (80/20% [v/v]). H_2_ + CO_2_ (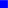) was determined by gas chromatography as described previously (Bertsch and Müller, 2015). OD_600_ (●), pH (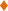) as well as acetate (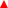), butyrate (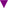) and isobutyrate (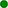) were determined as described in the legend to Fig. 2. B. *E. limosum* was grown at 37°C in 500 ml of phosphate buffered basal medium (Chang *et al*., 1999) with overpressure of 1 bar CO (100%) (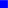). CO was determined by gas chromatography as described previously (Bertsch and Müller, 2015). OD_600_ (●), pH (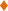) as well as acetate (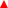), butyrate (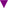), isobutyrate (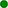) and ethanol (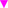) were determined as described in the legend to Fig. 2 (Bertsch and Müller, 2015). C. *E. limosum* KIST612 was grown at 37°C in 500 ml of CBBM with 20 mM Na^+^‐Formate (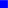) under a N_2_/CO_2_ (80/20% [v/v]) atmosphere. The concentration of formate was determined by a formate assay kit (R‐Biopharm, Pfungstadt, Germany). OD_600_ (●), pH (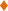) as well as acetate (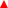), butyrate (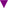) and isobutyrate (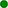) were determined as described in the legend to Fig. 2. All data points are mean ± SEM; *N* = 3 independent experiments.

The WLP accepts different C1 substrates with different oxidation/reduction states. Growth of *E*. *limosum* KIST612 on H_2_ + CO_2_ and CO have been described (Chang *et al*., 2001); the entry points are shown in Fig. 1. Formate is an intermediate of the WLP but has not been described as substrate for *E*. *limosum* KIST612. Growth on H_2_ + CO_2_ or CO proceeded with growth rates of 0.04 and 0.05 h^−1^; acetate was the major product, butyrate was only observed in trace amounts (Fig. 3). Cells also grew on formate with a growth rate similar to H_2_ + CO_2_ or CO (0.03 h^−1^) but the final yield was much lower (OD_600_ = 0.25). Growth was accompanied by acetate production, but due to the consumption of formate, in sum, the pH increased slightly. Butyrate was not produced.

### Growth with and product formation from methanol

**Fig. 4:**
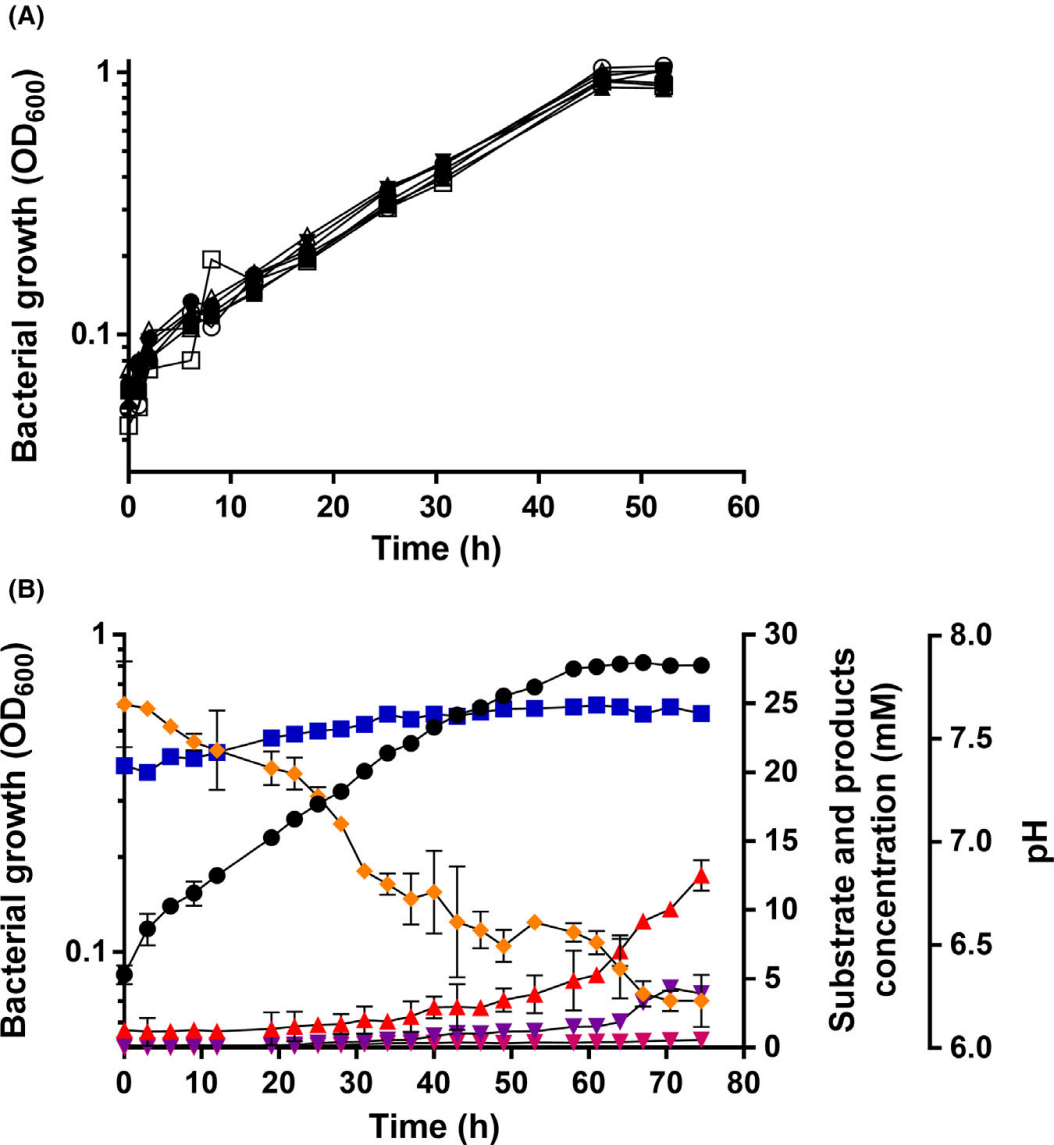
Growth of *E*. *limosum* on methanol. *E. limosum* KIST612 was grown at 37°C in 500 ml of CBBM with methanol under a N_2_/CO_2_ (80/20% [v/v]) atmosphere. A. Growth of *E*. *limosum* on 20 (●), 40 (○), 80(▼), 100 (▵), 120 (▲), 160 (■) or 200 (□) mM methanol. B. *E. limosum* KIST612 was grown at 37°C in 500 ml of CBBM with 20 mM methanol under a N_2_/CO_2_ (80/20% [v/v]) atmosphere. OD_600_ (●), pH (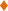) as well as acetate (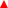), butyrate (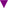), isobutyrate (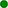), ethanol (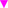) and methanol (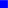) were determined as described in the legend to Fig. 2. All data points are mean ± SEM; *N* = 3 independent experiments

*E. limosum* did grow on methanol as sole carbon and energy source, as predicted from its genome sequence (Roh *et al*., 2011). Whereas *A. woodii* reached the maximum growth rate at 60 mM methanol (Kremp *et al*., 2018), growth of *E. limosum* was already maximal at 20 mM methanol. When a methanol‐adapted culture (two transfers) of *E. limosum* KIST612 was transferred to CBBM with 20 mM methanol, growth started immediately with a doubling time of 17.61 h and proceed for about 60 h, before the stationary phase started (Fig. 4). Parallel to growth, the methanol concentration dropped continuously with a rate of 0.29 mmol l^−1^∙h^−1^ to a residual concentration of 3.4 mM. Methanol consumption was accompanied by a production of acetate that reached a final concentration of 12 mM, corresponding to an acetate:methanol ratio of 0.53:1. Since acetogenesis from methanol according to Equation (1):

(1) $$4{\text{CH}}_{3}\text{OH}+2{\text{CO}}_{2}\to 3{\text{CH}}_{3}\text{COOH}+2H_{2}O\;\Delta G^{o^{\prime }}=‐212\;\text{kJ}\;{\text{mol}}^{‐1}$$

removes the CO_2_ from the solution leading to alkalinization parallel to acidification by acid production, the pH did not drop but increased slightly. Most important, again at the end of the exponential growth phase butyrate was produced and butyrate production reached a steady state at around 67 h. The final butyrate concentration was 3.7 mM, corresponding to a butyrate:methanol ratio of 0.17:1. Ethanol was not observed but traces of isobutyrate (0.5 mM). In sum, almost all substrate carbon (methanol + CO_2_) was recovered in the major end products acetate and butyrate (99 ± 10.3%).

## Conclusion

Here we describe for the first time that the acetogen *E. limosum* KIST612 produces butyrate from methanol. Growth on methanol requires the action of a methyltransferase system that transfers the methyl group from methanol to tetrahydrofolate and *E. limosum* KIST612 has a gene cluster similar to a previously suggested methanol‐specific methyltransferase system of *A. woodii* (Kremp and Müller, 2020). Buytrate production from acetyl‐CoA follows the pathways described for example for *Clostridium acetobutylicum* (Dürre *et al*., 2002) involving thiolase, hydroxybutyryl‐CoA dehydrogenase, crotonase and butyryl‐CoA dehydrogenase with the exception, that the latter is electron‐bifurcating and reduces ferredoxin alongside with crotonyl‐CoA (Jeong *et al*., 2015). As in clostridia, butanol could be produced from butyryl‐CoA by two subsequent reduction steps (Dürre, 2016). With the establishment of first genetic methods from *E. limosum* KIST612 (Jeong *et al*., 2020) it should be possible in the future to express butyryl‐CoA dehydrogenases in *E. limosum* KIST612. This would make methanol a promising feedstock for acetogenic production of butyrate.

## Conflict of interest

The authors declare no conflict of interest.
